# An *Apo-14* Promoter-Driven Transgenic Zebrafish That Marks Liver Organogenesis

**DOI:** 10.1371/journal.pone.0022555

**Published:** 2011-07-22

**Authors:** Rui Wang, Zhi Li, Yang Wang, Jian-Fang Gui

**Affiliations:** 1 State Key Laboratory of Freshwater Ecology and Biotechnology, Institute of Hydrobiology, Chinese Academy of Sciences, Wuhan, China; 2 Wuhan Center for Developmental Biology, Wuhan, China; 3 Graduate School of the Chinese Academy of Sciences, Beijing, China; Texas A&M University, United States of America

## Abstract

Several transgenic zebrafish lines for liver development studies had been obtained in the first decade of this century, but not any transgenic GFP zebrafish lines that mark the through liver development and organogenesis were reported. In this study, we analyzed expression pattern of endogenous *Apo-14* in zebrafish embryogenesis by whole-mount *in situ* hybridization, and revealed its expression in liver primordium and in the following liver development. Subsequently, we isolated zebrafish *Apo-14* promoter of 1763 bp 5′-flanking sequence, and developed an *Apo-14* promoter-driven transgenic zebrafish *Tg*(*Apo14: GFP*). And, maternal expression and post-fertilization translocation of *Apo-14* promoter-driven GFP were observed in the transgenic zebrafish line. Moreover, we traced onset expression of *Apo-14* promoter-driven GFP and developmental behavior of the expressed cells in early heterozygous embryos by out-crossing the *Tg*(*Apo14: GFP*) male to the wild type female. Significantly, the *Apo-14* promoter-driven GFP is initially expressed around YSL beneath the embryo body at 10 hpf when the embryos develop to tail bud prominence. In about 14-somite embryos at 16–17 hpf, a typical “salt-and-pepper” expression pattern is clearly observed in YSL around the yolk sac. Then, a green fluorescence dot begins to appear between the notochord and the yolk sac adjacent to otic vesicle at about 20 hpf, which is later demonstrated to be liver primordium that gives rise to liver. Furthermore, we investigated dynamic progression of liver organogenesis in the *Tg*(*Apo14: GFP*) zebrafish, because the *Apo-14* promoter-driven GFP is sustainably expressed from hepatoblasts and liver progenitor cells in liver primordium to hepatocytes in the larval and adult liver. Additionally, we observed similar morphology between the liver progenitor cells and the GFP-positive nuclei on the YSL, suggesting that they might originate from the same progenitor cells in early embryos. Overall, the current study provides a transgenic zebrafish line that marks the through liver organogenesis.

## Introduction

Cell fate tracing and tissue-specific transgenic imaging have highlighted some significantly conserved processes essential for vertebrate endoderm development and organ formation [1]. In zebrafish, several transgenic lines of green fluorescent protein (GFP) expression had been generated to emerge morphogenesis of digestion system. For example, the gutGFP zebrafish line *Tg(gutGFP)^s854^*, using a *Xenopus EF-1α* promoter to drive GFP expression in endoderm and endoderm-derived organs including liver, gut, and pancreas from about 30 hpf (hours post fertilization) through to adulthood, had already provided significant advantages for investigating morphogenesis of liver and pancreas [2], [3] and for revealing functional role of endothelial cells in later stages of the organogenesis[4]–[6]. The intestine-specific transgenic zebrafish that utilizes intestine-type fatty acid binding protein promoter to drive GFP expression was also generated, but the GFP-reporter gene expression was not observed until 72 hpf [7]. The *nkx2.2a* promoter-driven transgenic zebrafish line *Tg(nkx2.2a∶mEGFP)* could distinguish enteroendocrine cells first at 52 hpf in the caudal region of intestine[8]. With the Ds-Red RFP reporter gene under a liver-type fatty acid binding protein promoter, the liver growth phase was divided three distinct stages, such as avascular growth between 50–55 hpf, endothelium-dependent growth between 55–72 hpf and blood circulation-dependent growth after 72 hpf [9]. As far as we know, however, no any transgenic GFP zebrafish lines that mark liver, intestine, or pancreas development and morphogenesis from the onset expression of early endodermal cells in the gastrula embryos to the complete organ formation in the larvae have been obtained.

Apolipoproteins (Apo), which were known to function in lipid transport, uptake and homeostasis in vertebrates, have been recently suggested to play significant roles during early development. *Apo-14*, the 14 kDa apolipoprotein gene, was firstly cloned in eel (*Anguilla japonica*) [10], and also identified from at least 14 species of teleost fish [11]. Importantly, fish Apo-14 was shown to be the homologue of mammalian ApoA-II, because phylogenetic analysis revealed 27–58% sequence similarity between internal repeats of fish Apo-14 and mammalian ApoA-II [12]. As a fundamental constituent of vertebrate high density lipoproteins (HDLs), Apo-14 should have more significant functional roles. In hermaphroditic orange-spotted grouper (*Epinephelus coioides*) [13] and polyploid gibel carp (*Carassius auratus gibelio*) [14], *Apo-14* was demonstrated to be highly expressed during embryogenesis. Its transcript firstly appears in YSL (yolk syncytial layer) of early gastrula embryos at a high level, and subsequently concentrates to the digestive system of later embryos and early larvae[15], [16]. Morpholino knockdown of Apo-14 in gibel carp resulted in severe disruption of digestive organs and affected yolk lipid transportation and utilization. The data suggested that *Apo-14* should be required for digestive system organogenesis during embryogenesis and early larval development [16]. Therefore, *Apo-14* should be a potential marker to trace and understand morphogenesis and organogenesis of digestive system, especially liver.

To better trace developmental process of liver, we isolated zebrafish *Apo-14* promoter, and constructed an *Apo-14* promoter-driven transgenic zebrafish *Tg*(*Apo14: GFP*). Significantly, the *Apo-14* promoter-driven GFP was early expressed in YSL at about 10 hpf, then appeared as a small cluster of cells in liver primordium that gives rise to liver at about 20 hpf, and ultimately restricted to liver in later embryos and adults. Through this way, we traced the onset expression of *Apo-14* promoter-driven GFP and the developmental behavior of the expressed cells in the early heterozygous embryos by out-crossing the *Tg*(*Apo14: GFP*) male to the wild type female, and observed the dynamic progression of liver morphogenesis and organogenesis from the early endodermal cells to the complete organ formation.

## Results

### Endogenous *Apo-14* expression pattern in zebrafish embryos and larvae

**Figure 1:**
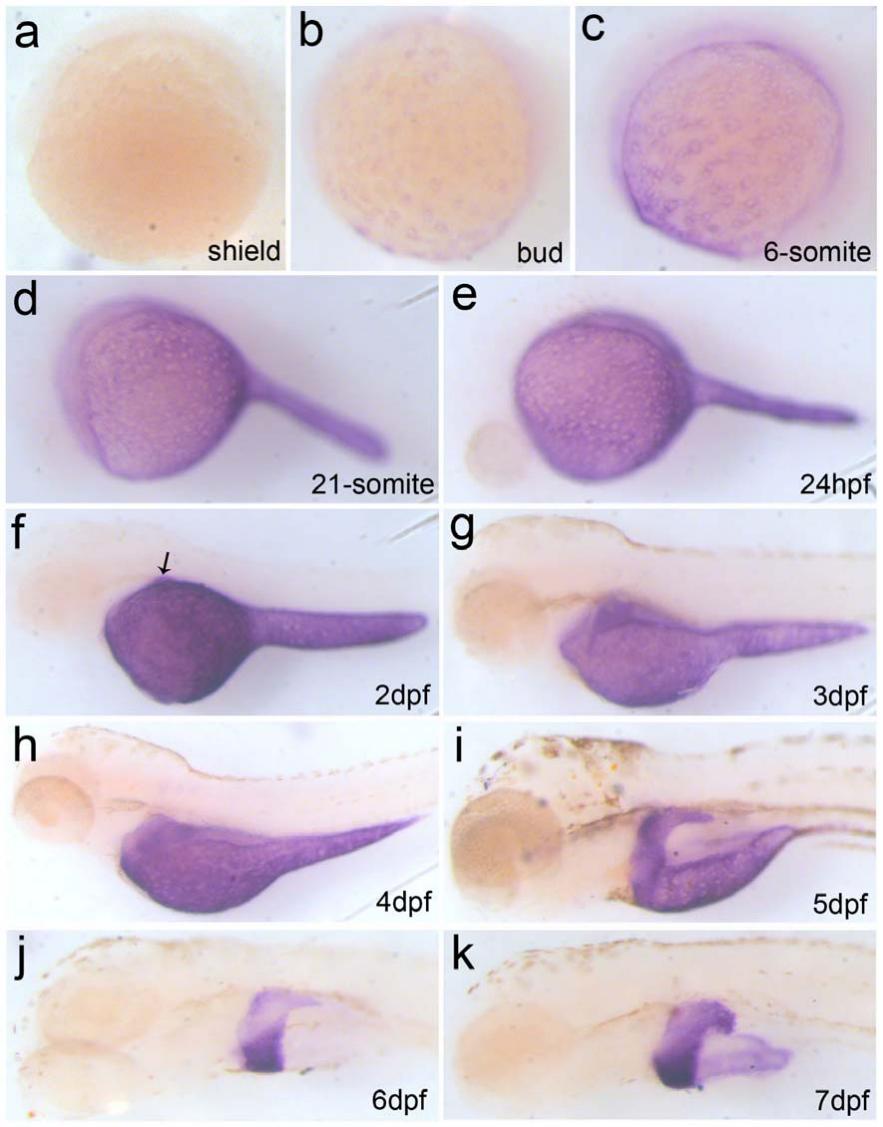
Expression pattern of endogenous *Apo-14* during zebrafish embryogenesis by whole-mount *in situ* hybridization. (a) shield (6 hpf); (b) bud (10 hpf); (c) 6-somite (12 hpf); (d) 21-somite(20 hpf); (e) 24 hpf; (f)2 dpf larva; (g) 3 dph larva; (h) 4 dph larva; (i) 5 dph larva; (j) 6 dph larva; (k) 7 dph larva. (a) dorsal to the right; (b–k) anterior to the left. The arrow indicates positive signals for the liver primordium.

Firstly, we analyzed expression pattern of endogenous *Apo-14* in zebrafish embryogenesis by whole-mount *in situ* hybridization. As shown in Figure1, endogenous *Apo-14* is initially expressed in YSL of embryos from bud stage at 10 hpf (Figure1b), and a similar “salt-and-pepper” expression pattern is obviously displayed in YSL of the early embryos from bud stage to 24 hpf (Figure 1b–e). The expression level increases continuously, and culminates at 2dpf in YSL (Figure 1f). Then, its transcript content decreases gradually after the following stages (Figure 1g–i), and almost consumes from the yolk sac at 6 dpf (Figure 1j). Significantly, the *Apo-14* transcript begins to appear in liver primordium at 2 dpf (Figure 1f), and accumulates with the liver morphogenesis (Figure 1f–k). The data suggested that zebrafish *Apo-14* should be a significant marker to trace morphogenesis and organogenesis of liver.

### Isolation and activity analysis of *Apo-14* promoter

**Figure 2:**
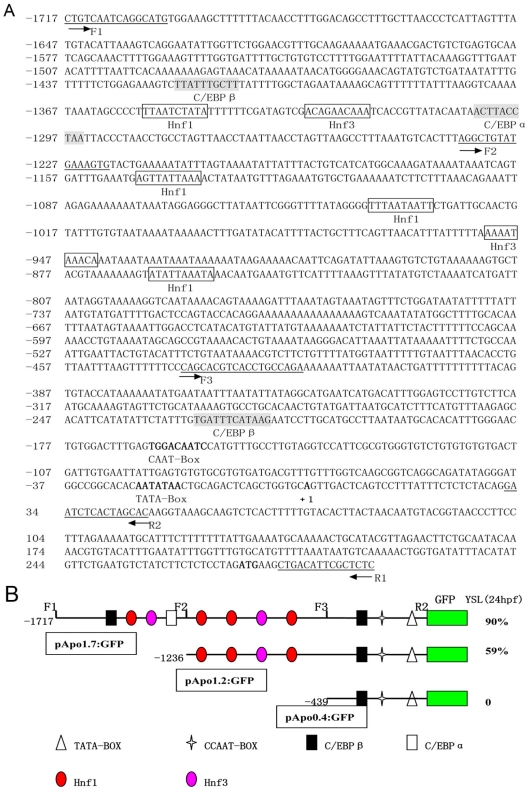
Characterization and activity analysis of zebrafish *Apo-14* promoter. (**A**) The 2008 bp promoter sequence of *Apo-14*. The numbers of the nucleotides positions are indicated on the left. Potential Hnf and C/EBP binding sites are boxed and shaded respectively. The putative TATA box and CAAT box, the transcription start site and the ATG start codon are bolded. The primer sequences are underlined and the arrows depict the positions and extension directions of the cloned sequence for construction excluding the added restriction site. (**B**) Schematic map of pApo1.7∶GFP, pApo1.2∶GFP and pApo0.4∶GFP and summary of GFP expression in transient expression assay. The series of promoter deletion constructs were made by forward primers at −1717 bp(F1), −1236 bp(F2), −439 bp(F3), and reverse primer at +46 bp(R2) as indicated, respectively. Important sequences are indicated by different shape blocks with different colors. These chimeric DNA constructs were injected into zebrafish embryos, and GFP expression was evaluated at 24 hpf. The percentages of injected embryos expressing GFP in the YSL are shown.

To determine regulatory sequence of zebrafish *Apo-14* promoter, we isolated a 2008 bp 5′-flanking region sequence of *Apo-14* from zebrafish genome DNA. Using the AliBaba2 program, many putative transcription factor binding sites including six consensus motifs for Hnf (at −855/−864, −943/−952, −1031/−1040, −1137/−1146, −1321/−1330 and −1346/−1355) and three consensus motifs for C/EBP (at−218/−227, −1295/−1304, and −1411/−1420) were found in the upstream region (Figure2A). And, a TATA-Box sequence (−20/−26) and a CAAT-Box sequence (−156/−164) were observed from the immediate upstream region (−20/−164) of the transcription start site, suggesting that the core promoter for *Apo-14* might be located around this region (Figure 2A).

To analyze the activity of *Apo-14* promoter, three fragments including 1763 bp (from +46 to −1717), 1282 bp (from +46 to −1236) and 485 bp (from +46 to −439) of the *Apo-14* 5′-flanking sequence were cloned into vector Tol2 with primers F1, F2, F3 and R2 (Table 1) as described previously[17], [18]. The constructs were respectively injected into one-cell embryos, and their activities were evaluated by the percentage of GFP-expressing embryos through transient transgenic assay at 24 hpf. As shown in Figure2B, up to 90% of pApo1.7∶GFP-injected embryos display strongly green fluorescence in YSL, and the construct pApo1.2∶GFP retains low ability (59%) to direct GFP expression in YSL, whereas the pApo0.4∶GFP construct completely loses the expression ability. The data indicate that the 1763 bp sequence from +46 to −1717 is needed for the *Apo-14* promoter activity.

**Table 1:** Primers used for all the experiments.

Name	Sequence (5′–3′)
apo14F	ATGAAGCTGACATTCGCTCTCAT
apo14R	TAATACGACTCACTATAGGGGCTGGACGGGATGTTATTTTC
F1	CTGTCAATCAGGCATG
R1	GAGAGCGAATGTCAG
R2	CTCGAGGTGCTAGTGAGATTC
F2	GGTACCAGGCTGTATGAAAGTG
F3	GGTACCCAGCACGTCACCTGCCAGA

### Generation of transgenic zebrafish line *Tg*(*Apo14: GFP*)

Subsequently, the larvae with GFP signal were raised to adulthood, and a total of 20 adults were respectively mated to wild type zebrafish. As a result, three founder individuals (15%) with GFP expression in liver were screened through germline transmission, and they were composed of two females and one male. The ratio of GFP-positive embryos among their offspring of the three founders ranged from 20% to 50%, presumably due to mosaicism of transgene in cells of the germ line. As the positive females were mated with the positive male, about 25% embryos appeared strongly green fluorescence. Moreover, the stable and homozygous *Apo-14* promoter-driven transgenic zebrafish line was conveniently established in F2, and nominated as *Tg*(*Apo14: GFP*). Significantly, when the transgenic zebrafish line was out-crossed to wild type, about 50% of their offspring displayed green fluorescence, indicating that only one single integration site exists in the transgenic zebrafish line although the possibility of multiple integration sites in a close chromosome region cannot be ruled out.

### Maternal expression and post-fertilization translocation of *Apo-14* promoter-driven GFP in transgenic zebrafish line *Tg*(*Apo14: GFP*)

**Figure 3:**
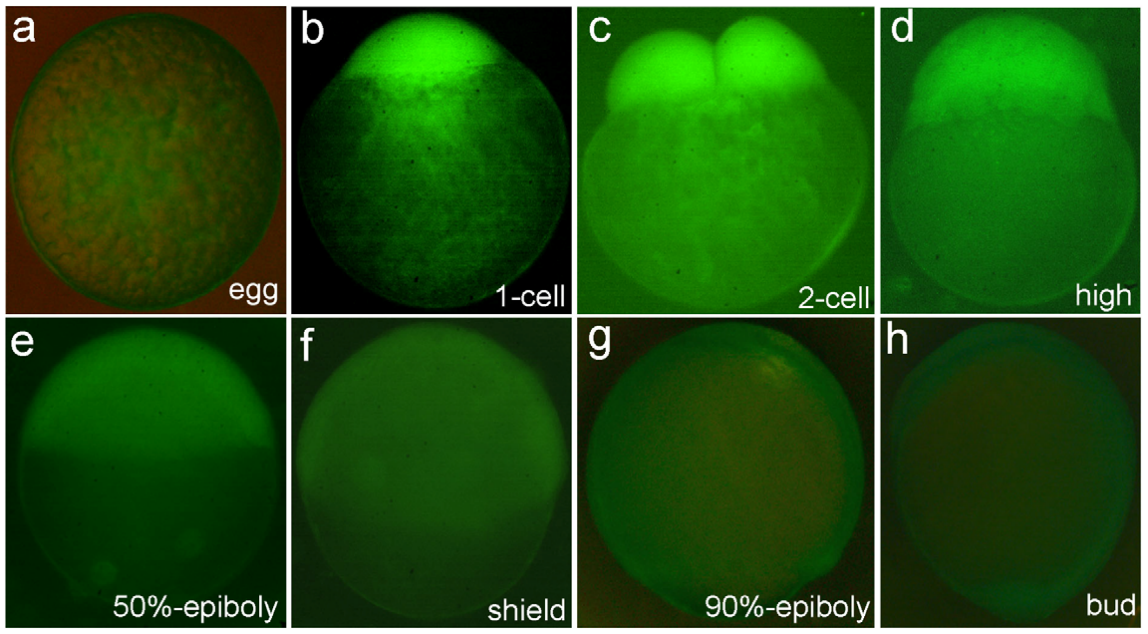
Observation of maternal expression and post-fertilization translocation of *Apo-14* promoter-driven GFP in transgenic zebrafish line *Tg*(*Apo14: GFP*). (a) unfertilized egg; (b) 1-cell embryo; (c) 2-cell embryo; (d) high blastula embryo; (e) 50%-epiboly embryo; (f) shield embryo; (g) 90%-epiboly embryo; (h) bud stage embryo.

Identical to the presence of maternal Apo-14 in gibel carp [16], the *Apo-14* promoter-driven GFP was also expressed in mature eggs of the *Tg*(*Apo14: GFP*) zebrafish, and uniformly distributed in the yolk (Figure3a). After the egg was fertilized, the GFP moved to animal pole along with the cytoplasm stream (Figure3b). Following the cleavage progress, the maternal GFP was equally distributed to the blastomeres (Figure3c–g), and gradually disappeared until the bud stage (Figure3h).

### Onset expression and developmental behavior of *Apo-14* promoter-driven GFP in early heterozygous embryos

**Figure 4:**
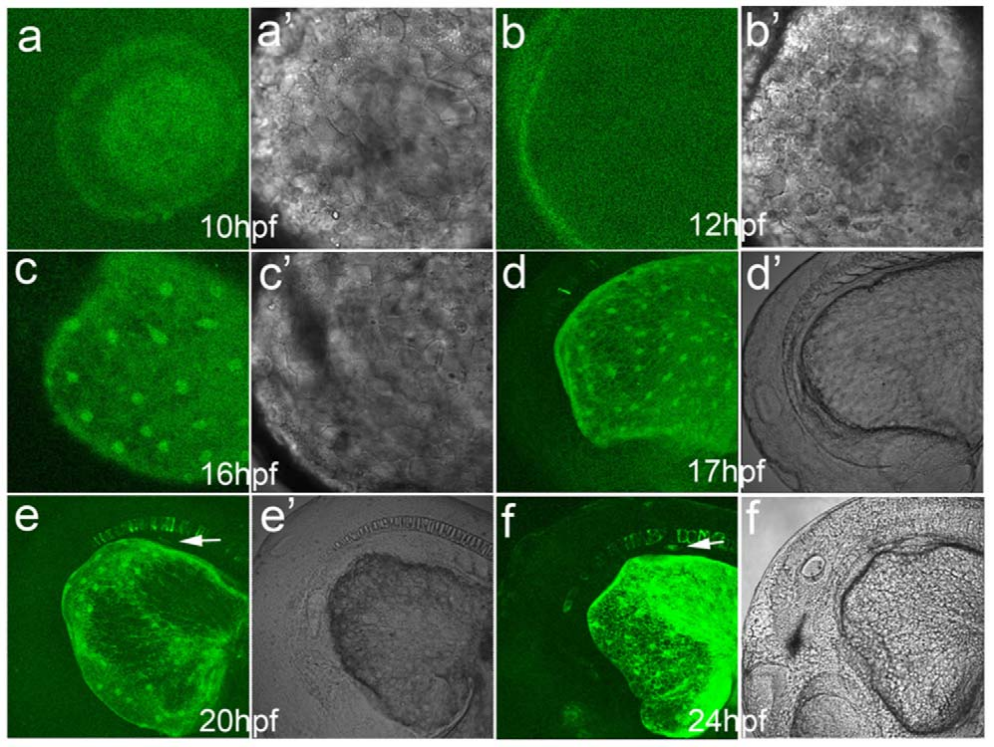
Onset expression and developmental behavior of *Apo-14* promoter-driven GFP in early heterozygous embryos produced by out-crossing the *Tg*(*Apo14: GFP*) male to the wild type female. (a, a’) bud embryo at 10 hpf; (b, b’) 5-somite embryo at 12 hpf; (c, c’) 14-somite embryo at 16 hpf; (d, d’) 17 hpf embryo; (e, e’) 20 hpf embryo; (f, f’) 24 hpf embryo. Arrows point to the liver primordium. The images (a–f) show the green fluorescence, and (a’–f’) the corresponding bright images.

To clarify the onset expression of *Apo-14* promoter-driven GFP and thereby to trace the developmental behavior of the expressed cells in early embryos, we produced heterozygous embryos by out-crossing the *Tg*(*Apo14: GFP*) male to the wild type female, because the heterozygous embryos avoided maternal GFP, and were able to characterize the onset and progression of expression with confocal laser scanning microscope. As shown in Figure4, the earliest GFP fluorescence is initially observed around YSL beneath the embryo body at 10 hpf when the embryos develop to tail bud prominent (Figure4a), and the green fluorescence ring becomes obvious at 12 hpf when the embryos develop to 5-somite stage (Figure4b). In about 14-somite embryos at 16–17 hpf, a typical “salt-and-pepper” expression pattern is clearly observed in YSL around the yolk sac (Figure4c and 4d), and green fluorescence also appears in the notochord at 17 hpf. In comparison with the corresponding bright images (Figure4c’ and 4d’), the GFP expressed cells locate in YSL with an ordered distribution. At about 20 hpf, a green fluorescence dot begins to appear between the notochord and the yolk sac adjacent to otic vesicle (Figure4e and 4e’). Significantly, the dot exists continually as observed at 24 hpf (Figure4f), and becomes very obvious along with the following developmental progress. Additionally, eyes are also lightened by the *Apo-14* promoter-driven GFP at about 24 hpf.

### Dynamic progression of liver morphogenesis in the *Tg*(*Apo14: GFP*) line

**Figure 5:**
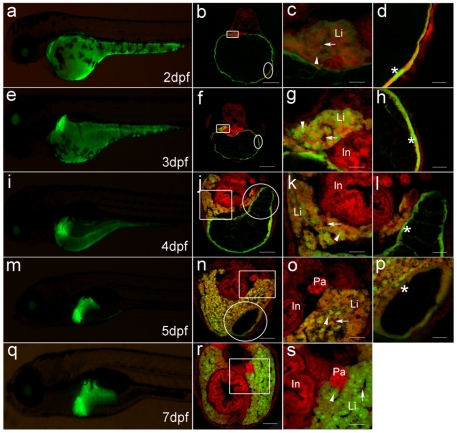
Dynamic GFP expression pattern in liver morphogenesis of *Tg(Apo14: GFP)* from 2dpf to 7dpf. (**a, e, i, m, q**) show the left lateral view of *Tg(Apo14: GFP)* larvae from 2dpf to 7dpf. (**b, f, j, n, r**) show the cross sections through intestine bulb and liver of the larvae at same stage as the left columns. Scale bar: 150 µm. (**c, g, k, o, s**) are magnifications of the corresponding frame areas in (b, f, j, n, r), which highlight the GFP expression in the hepatocytes. Scale bar: 30 µm. (**d, h, l, p**) are magnifications of the corresponding circle areas in (b, f, j, n), which highlight the GFP expression on the YSL. Scale bar: 30 µm. Green fluorescence is emitted from the *Apo-14* promoter-driven GFP, and red fluorescence is stained by PI for showing the nuclear position. Arrowheads point to hepatocytes, arrows mark sinusoids, and the asterisks delineate the GFP-expressed nuclei on YSL. Li: liver; In: intestine bulb; Pa: pancreas.

To make sure that the green fluorescence dot is liver primordium that will give rise to liver, we further observed the developmental fate through transverse sections. As shown in Figure5, the GFP-positive cells are observed in the triangle liver primordium on the left of 2 dpf embryos (Figure5a and 5b). In comparison with strong fluorescence of the spindly GFP-positive nuclei on the YSL, the positive cells in liver primordium appear weak fluorescence, and are very large and associate together without clear cell borders (Figure5c and 5d), which might be liver progenitor cells, the hepatoblasts. At 3dpf (Figure 5e), the GFP-positive cells in liver primordium become small, and the fluorescence intensity is same to that on the yolk sac (Figure 5f, 5g and 5h), indicating that the cells have differentiated into hepatocytes from hepatoblasts. Moreover, the sinusoid begins to form among the hepatocytes, and blood cells are observed in the nascent sinusoids (Figure5g). About 4dpf, along with reduction of yolk sac (Figure5i), the primitive liver obviously enlarges, and extends ventrally from the left to the right across the ventral side of the rostral region of intestinal tract (Figure5j and 5k). At that time, the left lobe of the liver is composed of multiple layers of hepatic cords and enlarged sinusoids (Figure5k), whereas the right lobe has only two layers of hepatic cords (Figure5l). The dramatic growth and size changes undergo at 5dpf (Figure5m), in which growth results in medial expansion between the two lobes (Figure5n and 5o). Noticeably, along with further reduction of yolk sac, the spindly GFP-positive nuclei on the YSL become closer with each other (Figure5p), and their morphology is very similar to the hepatoblasts in liver primordium of 2dpf embryos (Figure5c), indicating that they originate from the same progenitor cells, and play similar function in early embryos[19]. After 7dpf, the liver size and GFP expression continue to increase (Figure5q and 5r). At that time, the liver has been well organized (Figure5r and 5s), and yolk sac and YSL have completely disappeared. Significantly, the *Apo-14* promoter-driven GFP is expressed specifically in liver and YSL of digestion system, whereas no any positive signals are observed in other digestion organs, such as pancreas and intestine bulb.

**Figure 6:**
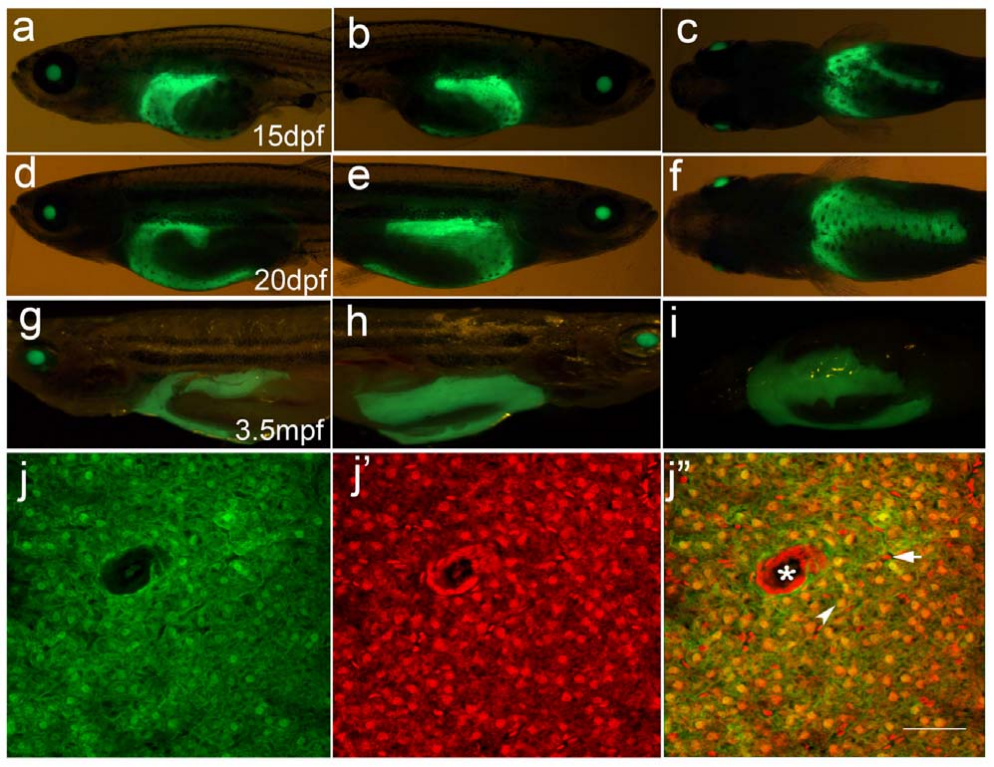
Outgrowth of the third lobe of liver from 15dpf to 20dpf and liver sections of adult fish of *Tg(Apo14: GFP)*. (**a**–**c**) 15 dpf larvae; (**d**–**f**) 20 dpf larvae; (**g**–**i**) 3.5 mpf adult fishes. (**a, d, g**) are the left lateral views showing left lobe of the liver. (**b, e, h**) are the right lateral views showing right lobe of the liver. (**c, f, i**) are the central views showing ventral lobe of the liver. Anterior is to the left. (**j, j’, j’’**) are the cross sections through the liver of 3.5 mpf adult fish. **(j)** shows the green GFP fluorescence in the hepatocytes, (**j’**) shows the red fluorescence stained by PI, (**j’’**) is the mergence of the green and red fluorescence. Arrowhead points to the hepatocyte, arrow marks sinusoid, and the asterisk delineates the central vien. Scale bar: 30 µm.

Around 15 dpf, the liver begins to outgrow from the ventral portion (Figure6a, 6b and 6c), and forms the third liver lobe at 20 dpf (Figure6d, 6e and 6f). At that time, the liver is composed of three lobes: left, right and ventral. Even in the adults, the three liver lobes are still visible from the body surface (Figure 6g, 6h and 6i). Cellular observation of adult liver tissue indicates that the *Apo-14* promoter-driven GFP is still expressed by the hepatocytes, whereas the sinusoidal endothelial cells that constitute capillaries or veins do not express GFP (Figure 6j).

## Discussion

Here we showed that an approximately 1.7-kb 5′-upstream region of *Apo-14* contained sufficient regulatory elements to direct tissue-specific expression of GFP throughout the zebrafish life cycle. It is the first time to clone *Apo-14* regulatory sequence and reveal its putative transcription factor binding sites, such as four Hnf1, two Hnf3 and three C/EBP binding sites, which concur with GFP expression pattern in transgenic fish. Following the GFP expression in *Tg(Apo14: GFP)*, we observed the maternal expression pattern. Using the heterozygous embryos by out-crossing the *Tg*(*Apo14: GFP*) male to the wild type female, we could trace the onset expression of *Apo-14* promoter-driven GFP and the developmental behavior of the expressed cells in YSL, and observed the dynamic progression of liver morphogenesis from the onset expression of early endodermal cells to the complete organ formation. Obviously, the expression pattern recapitulates that of *Apo-14* in orange-spotted grouper [15] and in gibel carp embryogenesis[16], indicating that the *Apo-14* promoter drives reliably reporter gene expression in an typical manner identical to the endogenous gene.

The onset expression of GFP in *Tg(Apo14: GFP)* transgenic line is reminiscent of the spatiotemporal expression of ceruloplasmin (*cp*), the earliest liver differentiation marker described so far in zebrafish, which was reported to be asymmetrically expressed on the left hand side of the endoderm at 16 hpf (14-somite stage), and in the developing liver by 32h pf[20]. Additional *cp* expression was detected in a spotty pattern in the ‘yolk sac’ and this spotty expression became reduced specifically on the left side at the time of liver formation. This finding led the investigators to suggest that some endodermal cells resided in the ‘yolk sac’ might migrate towards the developing liver and subsequently contribute to liver development[20]. However, the presence of endodermal cells in the ‘yolk sac’ was not detected by the established endodermal markers, such as foxA1–A3[21] or by the lineage analysis[22]. In addition, no any evidence for the endodermal cell migration from the ‘yolk sac’ to the developing liver was shown in the gutGFP line, a stable transgenic line that expresses GFP throughout the developing digestive system [3]. In this study, we have observed that the *Apo-14* promoter-driven GFP is expressed in YSL early at 10 hpf when the embryos just develop to tail bud prominent, and then presented as a typical “salt-and-pepper” expression pattern in YSL around the yolk sac. At about 20 hpf, a green fluorescence dot, which was late demonstrated as liver primordium, was observed, and its detailed dynamic progression of liver morphogenesis was recorded along with the embryogenesis. Significantly, the *Apo-14* promoter-driven GFP is sustainably expressed from the hepatoblasts, the liver progenitor cells in embryo liver primordium to the hepatocytes in larval and adult liver of the *Tg*(*Apo14: GFP*) zebrafish, and similar morphology between the hepatoblasts and the GFP-positive nuclei on the YSL was also observed.

Apolipoproteins have been demonstrated to play physiological roles on nutrition transport and energy metabolism, and numerous variants have been revealed in some apolipoproteins including ApoA1, ApoA5, ApoB, ApoC3 and ApoE[23]–[25], which are associated with the risk of cardiovascular disease [26]. And, most apolipoproteins are expressed in liver. Apparently, the *Tg*(*Apo14: GFP*) zebrafish line should be a potential tool for understanding the physiological function and the signaling molecules of apolipoproteins.

## Materials and Methods

### Fish maintenance

Zebrafish were maintained in the aquarium of Freshwater Ecology and Biotechnology Laboratory with a controlled light cycle of 14 h light/10 h dark at 28°C. All embryos were collected by natural spawning and staged according to morphological criteria[27]. The animal protocol for this research was approved by the Institute of Hydrobiology Institutional Aimal Care and Use Committee (Approval ID: keshuizhuan 0829).

### Whole-mount *in situ* hybridization

Embryos were fixed in 4% (w/v) paraformaldehyde according to a previous report [28]. The fragment amplified by RT-PCR from zebrafish liver cDNA library with the primers apo14F and apo14R (Table 1) which included T7 RNA polymerase binding sequence at the 3′ end. Antisense digoxigenin-UTP labeled RNA probe was generated using T7 polymerase by *in vitro* transcription (DIG RNA labeling kit; Roche Molecular Biochemicals). The procedure of whole-mount *in situ* hybridization was performed as described previously [28].

### Promoter cloning and construct preparation

The zebrafish genomic DNA was isolated from the fins of zebrafish by standard phenol/chloroform extraction. Based on zebrafish genome sequence (GenBank accession no. DF003626), the promoter containing 2008 bp upstream of the 5′-flanking region was firstly isolated with specific primers F1 and R1 (Table 1) and sequenced completely by Bioasia sequencing company. Potential Hnf and C/EBP binding sites were analyzed by the AliBaba2 program (www.gene-regulation.com). The TATA box and CCAAT box, the transcription start site and the ATG start codon were predicted by the software from the network (http://www.fruitfly.org/seq_tools/promoter.html). A proximal promoter containing 1763 bp upstream near the transcription start site was cloned with primer F1 and R2 (Table 1), then inserted into Tol2 vector. The resulted DNA construct was named pApo1.7: GFP. To make deletion constructs, 1262 bp and 485 bp fragments was amplified with upstream primers F2, F3 and downstream primer R2 (Table 1) and ligated into Tol2 vector between the restriction sites XhoI and KpnI as described previously[28]–[30]. These deleted constructs included 1262-bp, 485-bp 5′- flanking region of *Apo-14* were referred to pApo1.2: GFP and pApo0.4: GFP respectively. All three constructs were injected into one-cell stage zebrafish embryos respectively and the GFP expression was observed at 24 hpf.

### Production of transgenic zebrafish

To generate the transgenic lines, transposase mRNA was synthesized *in vitro* [31]. The pApo1.7:GFP was dissolved in water at the concentration of 200 ng/µl. Then the construct was co-injected with the transposase mRNA into zebrafish 1-cell embryos as reported previously[32]. The injected embryos were incubated at 28°C, and the transient expression pattern of *Apo-14* promoter-driven GFP similar to that of endogenous *Apo-14* was observed in the pApo1.7∶GFP-injected zebrafish embryos and larvae. This implied that the 1763 bp *Apo-14* promoter sequence might contain all necessary cis-elements for tissue-specific expression in zebrafish, and the pApo1.7: GFP construct could be used to make the stable transgenic zebrafish line.

GFP-positive fish were raised to sexual maturity and crossed with wild type zebrafish. Germline integrated transgenic zebrafish were screened and progeny were saved and referred to F1. The GFP expression of embryos from F1 transgenic zebrafish was examined for further analysis. The successive progeny expressing GFP were raised and named in proper order for further analysis and breeding. The transgenic zebrafish lines was generated by pApo1.7: GFP and named *Tg(Apo14: GFP)*.

### GFP fluorescence observation

For analyzing GFP fluorescent patterns, embryos were anesthetized with 30 mg/ml methanesulfonate salt (Sigma). High magnification images of early embryos were obtained by a Leica confocal laser scanning microscope. Low magnification images of larva were taken using a GFP filter on the stereomicroscope (Leica, Germany).

For tissue section, *Tg(Apo14: GFP)* larvae of 2 dpf, 3 dpf, 4 dpf, 5 dpf, 7 dpf and the liver of adult fish were fixed with 4% paraformaldehyde, washed with phosphate-buffered saline (PBS), immersed in 30% sucrose, frozen in O.C.T (Optimal Cutting Temperature, Germany) and sectioned at 10 µm in thickness with frozen microtomy (Leica). The cryostat sections were rehydrated in PBS for 30 minutes, stained by propidium iodide (PI, 5 µg/ml), washed 3 times with PBS (10 minutes each), viewed finally with fluorescence optics of Confocal Microscope (Leica) as described [33], [34].

## References

[1] Zorn AM, Wells JM (2009). Vertebrate Endoderm Development and Organ Formation.. Annu Rev Cell Dev Biol.

[2] Field HA, Dong PDS, Beis D, Stainier DYR (2003). Formation of the digestive system in zebrafish. II. pancreas morphogenesis.. Dev Biol.

[3] Field HA, Ober EA, Roeser T, Stainier DYR (2003). Formation of the digestive system in zebrafish. I. liver morphogenesis.. Dev Biol.

[4] Ober EA, Verkade H, Field HA, Stainier DYR (2006). Mesodermal Wnt2b signalling positively regulates liver specification.. Nature.

[5] Dong PDS, Munson CA, Norton W, Crosnier C, Pan X (2007). Fgf10 regulates hepatopancreatic ductal system patterning and differentiation.. Nat Genet.

[6] Chung W-S, Andersson O, Row R, Kimelman D, Stainier DYR (2010). Suppression of Alk8-mediated Bmp signaling cell-autonomously induces pancreatic β-cells in zebrafish.. Proc Natl Acad Sci U S A.

[7] Her GM, Chiang CC, Wu JL (2004). Zebrafish intestinal fatty acid binding protein (I-FABP) gene promoter drives gut-specific expression in stable transgenic fish.. Genesis.

[8] Ng ANY, de Jong-Curtain TA, Mawdsley DJ, White SJ, Shin J (2005). Formation of the digestive system in zebrafish: III. Intestinal epithelium morphogenesis.. Dev Biol.

[9] Korzh S, Pan X, Garcia-Lecea M, Winata C, Pan X (2008). Requirement of vasculogenesis and blood circulation in late stages of liver growth in zebrafish.. BMC Dev Biol.

[10] Kondo H, Kawazoe I, Nakaya M, Kikuchi K, Aida K (2001). The novel sequences of major plasma apolipoproteins in the eel *Anguilla japonica*.. Biochimica et Biophysica Acta (BBA) - Molecular and Cell Biology of Lipids.

[11] Kim K-Y, Cho YS, Bang I-C, Nam YK (2009). Isolation and characterization of the apolipoprotein multigene family in *Hemibarbus mylodon* (Teleostei: Cypriniformes).. Comp Biochem Physiol B Biochem Mol Biol.

[12] Choudhury M, Yamada S, Komatsu M, Kishimura H, Ando S (2009). Homologue of mammalian apolipoprotein A-II in non-mammalian vertebrates.. Acta Biochimica Et Biophysica Sinica.

[13] Zhou L, Gui J-F (2010). Molecular mechanisms underlying sex change in hermaphroditic groupers.. Fish Physiol Biochem.

[14] Gui J, Zhou L (2010). Genetic basis and breeding application of clonal diversity and dual reproduction modes in polyploid *Carassius auratus gibelio*.. Science China Life Sciences.

[15] Zhou L, Wang Y, Yao B, Li C-J, Ji G-D (2005). Molecular cloning and expression pattern of 14 kDa apolipoprotein in orange-spotted grouper, *Epinephelus coioides*.. Comp Biochem Physiol B Biochem Mol Biol.

[16] Xia JH, Liu JX, Zhou L, Li Z, Gui JF (2008). Apo-14 is required for digestive system organogenesis during fish embryogenesis and larval development.. Int J Dev Biol.

[17] Zhu R, Zhang YB, Zhang QY, Gui JF (2008). Functional domains and the antiviral effect of the double-stranded RNA-dependent protein kinase PKR from *Paralichthys olivaceus*.. J Virol.

[18] Jin JY, Zhou L, Wang Y, Li Z, Zhao JG (2010). Antibacterial and antiviral roles of a fish beta-defensin expressed both in pituitary and testis.. PLoS ONE.

[19] D'Amico LA, Cooper MS (2001). Morphogenetic domains in the yolk syncytial layer of axiating zebrafish embryos.. Dev Dyn.

[20] Korzh S, Emelyanov A, Korzh V (2001). Developmental analysis of ceruloplasmin gene and liver formation in zebrafish.. Mech Dev.

[21] Odenthal J, Nusslein-Volhard C (1998). fork head domain genes in zebrafish.. Dev Genes Evol.

[22] Warga R, Nusslein-Volhard C (1999). Origin and development of the zebrafish endoderm.. Development.

[23] Kathiresan S, Melander O, Guiducci C, Surti A, Burtt NP (2008). Six new loci associated with blood low-density lipoprotein cholesterol, high-density lipoprotein cholesterol or triglycerides in humans.. Nature Genet.

[24] Pollin TI, Damcott CM, Shen H, Ott SH, Shelton J (2008). A Null Mutation in Human APOC3 Confers a Favorable Plasma Lipid Profile and Apparent Cardioprotection.. Science.

[25] Willer CJ, Sanna S, Jackson AU, Scuteri A, Bonnycastle LL (2008). Newly identified loci that influence lipid concentrations and risk of coronary artery disease.. Nature Genet.

[26] Lusis AJ, Pajukanta P (2008). A treasure trove for lipoprotein biology.. Nature Genet.

[27] Kimmel CB, Ballard WW, Kimmel SR, Ullmann B, Schilling TF (1995). Stages of embryonic development of zebrafish.. Dev Dyn.

[28] Huang W, Zhou L, Li Z, Gui JF (2009). Expression pattern, cellular localization and promoter activity analysis of ovarian aromatase (Cyp19a1a) in protogynous hermaphrodite red-spotted grouper.. Mol Cell Endocrinol.

[29] Liu S, Li Z, Gui JF (2009). Fish-specific duplicated *dmrt2b* contributes to a divergent function through Hedgehog pathway and maintains left-right asymmetry establishment function.. PLoS One.

[30] Xie J, Farage E, Sugimoto M, Anand-Apte B (2010). A novel transgenic zebrafish model for blood-brain and blood-retinal barrier development.. BMC Dev Biol.

[31] Fisher S, Grice EA, Vinton RM, Bessling SL, Urasaki A (2006). Evaluating the biological relevance of putative enhancers using Tol2 transposon-mediated transgenesis in zebrafish.. Nat Protocols.

[32] Parinov S, Kondrichin I, Korzh V, Emelyanov A (2004). Tol2 transposon-mediated enhancer trap to identify developmentally regulated zebrafish genes *in vivo*.. Dev Dyn.

[33] Peng JX, Xie JL, Zhou L, Hong YH, Gui JF (2009). Evolutionary conservation of Dazl genomic organization and its continuous and dynamic distribution throughout germline development in gynogenetic gibel carp.. J Exp Zool B Mol Dev Evol.

[34] Sun M, Li Z, Gui JF (2010). Dynamic Distribution of Spindlin in Nucleoli, Nucleoplasm and Spindle From Primary Oocytes to Mature Eggs and its Critical Function for Oocyte-to-Embryo Transition in Gibel Carp.. J Exp Zool.

